# A Broad Mixture of Linear and Branched Perfluoroalkyl Substances (PFAS) in Hay: Results of an Interlaboratory Comparison

**DOI:** 10.1021/acs.jafc.5c09362

**Published:** 2026-02-09

**Authors:** Anne Jahnke, Konstantin Simon, Caroline Goedecke-Röber, Janine Kowalczyk, Michael Weiss, Anja Lüth

**Affiliations:** 27652 German Federal Institute for Risk Assessment, Department Safety in the Food Chain, Max-Dohrn-Str. 8-10, Berlin 10589, Germany

**Keywords:** interlaboratory comparison, poly- and perfluoroalkyl substances (PFAS), branched PFAS, hay, feed

## Abstract

Poly- and perfluoroalkyl substances (PFAS) are a class of compounds with significant economic impact and various applications since the 1950s. Many PFAS accumulate in the environment and pose a threat to consumers through their transfer along the food chain, and are, therefore, classified as persistent organic pollutants. In this interlaboratory comparison, nine laboratories analyzed hay, grown on a highly contaminated field in Germany, for its PFAS content. In addition, three of these nine laboratories quantified branched PFAS isomers individually. The results show that PFAS are present in high concentrations (up to 148 μg/kg based on 88% dry weight; dw), with a considerable contribution (31%) of branched PFAS in the hay. The study also highlights challenges in the quantification of branched PFAS due to the lack of commercially available standard solutions. The performance of the participating laboratories was excellent, with 93% of the results falling in a *z*-score range within the acceptable limits of |±2|.

## Graphical abstract

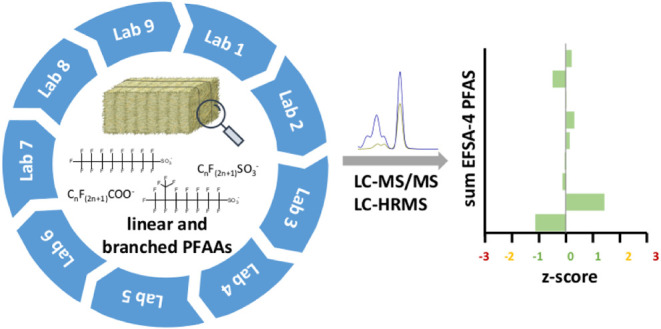

## 1 Introduction

Poly- and perfluoroalkyl substances (PFAS) are a class of compounds with significant economic impact and various applications.^1^ They are used in consumer products such as food packaging, cookware, and outdoor gear,^2,3^ have technical applications as refrigerants and sealing materials,^4^ and recently, fluorine-containing pharmaceuticals have been included as PFAS under a broadened Organization for Economic Co-operation and Development (OECD) definition.^5^ However, most of them are chemically extremely stable.^6–8^ Effluents from production and waste treatment can lead to contaminations in the environment.^9^ Another important source of contamination is aqueous film-forming foams (AFFF) containing PFAS, which are commonly used at airports, military bases, and firefighting departments.^10–12^

According to the OECD, PFAS are organic substances containing at least one fully fluorinated methyl or methylene carbon atom.^3^ This broad definition comprises thousands of anthropogenic chemicals, including positively and negatively charged, zwitterionic, and neutral substances.^3,13^ Most PFAS show unique properties such as persistence, resistance to acids and high temperatures, as well as hydrophobic and oleophobic properties.^14^ The perfluoroalkyl acids (PFAAs) are a class of PFAS that represent the perfluorosulfonic and perfluorocarboxylic acids. PFAAs are categorized by their chain length (C_*n*_F2_*n*_+1) into short- and long-chain substances. Perfluorocarboxylic acids with seven or more perfluorinated carbons and perfluorosulfonic acids with six or more perfluorinated carbons are defined as long-chain PFAS.^15^ The respective short-chain homologues are often termed as emerging PFAS, as they are frequently manufactured to replace the more strongly regulated long-chain PFAS, which have been in use for decades and for which more studies are available.^16^

Contamination and bioaccumulation of PFAS are critical because some PFAS are toxicologically active.^17^ Long-chain PFAS are linked to multiple adverse conditions, including decreased response to vaccines,^18–21^ thyroid disease,^22–26^ and nonalcoholic fatty liver disease.^22,27–29^ The main exposure routes for humans are food of animal origin,^30^ dust inhalation^31,32^ and exposure to consumer products^33^ as well as drinking water near contaminated sites.^34,35^ Therefore, knowledge on contamination is crucial to assess the risk of exposure to human beings.

It was shown that certain PFAS can transfer between environmental compartments.^36^ In addition, the contamination of soil can be caused by industrial emissions, the use of fire-fighting foams, and by fertilizers such as sewage sludge or compost.^37^ In southwest Germany (Rastatt; Baden-Württemberg) and west Germany (Brilon-Scharfenberg; North Rhine-Westphalia), for example, the use of compost mixed with paper sludge, deposited by farmers onto arable land, was likely to be the source of the contamination. In both regions, high concentrations of PFAS have been found in soil, plants, groundwater, and drinking water.^38,39^ As part of a monitoring program in Brilon-Scharfenberg, the total PFAS concentrations in the soil were found to exceed 6000 μg/kg.^40^ Investigations in the region of Rastatt have revealed that PFAS concentrations in the topsoil are highly variable, reaching up to 1000 μg/kg total PFAS, with individual cases exceeding this level.^41^ The contamination of agricultural land with PFAS can lead to these substances being taken up by plants intended for feed or food, which may then result in PFAS entering livestock.

Because of their environmental persistence and toxicological relevance, PFAS are being phased out by industry and law.^22^ PFOS, PFOA, and PFHxS (abbreviations: Table 1) are listed in Annexes A (PFOA, PFHxS) and B (PFOS) of the Stockholm Convention on persistent organic pollutants (POPs).^42^ Production, use, and trade are banned in the European Union.^43–45^ The European Chemicals Agency (ECHA) and the United States Department of Defense recommend banning PFAS in aqueous film-forming foams in the future.^46,47^

**Table 1:** Abbreviations of All Investigated PFAS and Respective Reported Content^a^

PFAS	lab 1	lab 2	lab 3	lab 4	lab 5	lab 6	lab 7	lab 8	lab 9	median	*σ*_rel_ [%]
Linear perfluorooctanesulfonic acid (PFOS)	—	64.13	55.00	60.00	64.03	65.31	61.00	53.00	75.00	62.52	11
Branched PFOS (br-PFOS)^b^	—	17.48	10.00	12.00	9.89	14.43	8.20	10.00	8.80	10.00	28
Total PFOS	54.00	90.47^c^	65.00	72.00	73.92	80.00	69.00	63.00	84.00	72.00	16
Linear perfluorooctanoic acid (PFOA)	48.00	78,59	64.00	59.00	61.55	60.47	63.00	46.98	63.00	61.55	15
Linear perfluorononanoic acid (PFNA)	0.22	0.45	—	0.22	0.30	0.30	0.35	0.24	—	0.30	28
Linear perfluorohexanesulfonic acid (PFHxS)	1.10	2.02	1.20	1.10	1.41	1.24	1.30	1.10	1.40	1.24	22
Sum EFSA-4 (lower bound)	103.00	171.53	130.00	132.00	137.18^e^	141.76	134.00	120.00	139.00^e^	134.00	14
Linear perfluorobutanesulfonic acid (PFBS)	0.51	0.55	0.48	0.48	0.56	0.56	0.61	0.49	0.72	0.55	14
Linear perfluoropentanesulfonic acid (PFPeS)	0.11	—	—	0.10	0.11	0.12	—	—	—	0.11	6
Linear perfluoroheptanesulfonic acid (PFHpS)	0.20	0.39	—	0.15	0.28	0.31	0.27	0.30	—	0.28	29
Linear perfluorononanesulfonic acid (PFNS)	44.00	74.05	—	23.00	49.08	47.48	—	42.90	90.00	47.48	42
Linear perfluorodecanesulfonic acid (PFDS)	99.00	141.04	—	67.00	116.43	86.68	61.00	93.5	145.00	96.25	31
Linear perfluorobutanoic acid (PFBA)	126.00	114.23	130.00	166.00	160.84	166.96	148.00	140.00	193.00	148.00	17
Linear perfluoropentanoic acid (PFPeA)	34.00	53.72	34.00	45.00	45.94	40.16	17.00	35.89	53.00	40.16	29
Linear perfluorohexanoic acid (PFHxA)	9.30	14.18	8.20	10.00	11.06	9.02	9.70	8.82	11.00	9.70	18
Linear perfluoroheptanoic acid (PFHpA)	2.40	3.81	3.00	2.90	3.12	2.83	2.50	2.50	3.20	2.90	15
Linear perfluorodecanoic acid (PFDA)	0.40	0.74	0.41	0.39	0.57	0.50	0.54	0.41	—	0.46	24
Linear perfluoroundecanoic acid (PFUnDA)	0.27	0.43	0.27	0.22	0.37	0.31	0.35	0.24	—	0.29	23
Linear perfluorododecanoic acid (PFDoDA)	0.40	0.87	—	0.54	0.64	0.52	0.58	0.63	—	0.58	24
Linear perfluorotridecanoic acid (PFTrDA)	0.20	1.09	—	0.28	0.29	0.44	—	0.24	—	0.29	79
Linear perfluorotetradecanoic acid (PFTeDA)	—	0.92	—	0.12	0.24	0.21	0.29	—	—	0.24	90
Total PFAS (calculated)										482.38	
Extraction	Acetonitrile^d^	Methanol	Methanol	Ion pair	Acetonitrile	Aacetonitrile^d^	Acetonitrile	Methanol	Ion pair	—	—
Cleanup	SPE	SPE	SPE	—	modified QuEChERS	modified QuEChERS	SPE	SPE	—	—	—

a Content reported as μg/kg based on 88% dry weight with extraction and clean up method . *σ*_rel_: relative standard deviation . Only the total PFAS content (μg/kg; 88% dry weight) of the shown isomers , Based on the median values , was calculated by the organizing laboratory .

b Sum of all branched isomers.

c The laboratory used a different method for the quantification of the total PFOS content.

d QuEChERS approach using buffer salts.

e Linear PFOS was used for the sum parameter.

The European Food Safety Authority (EFSA) concluded that food is the major route of exposure. For adults, 87.1%, and for children, 90.7% of the contribution comprises PFOS, PFOA, PFNA, and PFHxS, the so-called EFSA-4.^48^ These long-chain PFAAs have half-lives of several years in the human body, whereas short-chain alternatives are excreted more rapidly.^48^ Hence, EFSA established the tolerable weekly intake of 4.4 ng/kg body weight for the sum of EFSA-4 to assess potential effects due to long-term exposure to them.^48^

The toxicological impact of short-chain alternatives is not fully understood.^22^ Another category of scarcely studied PFAS is the branched PFAS (br-PFAS). These PFAS feature a branched alkyl chain and are byproducts of the widely applied electrochemical fluorination production method.^14,49,50^ It has been shown that branched PFAS accumulate differently than linear PFAS in the environment and living beings^51^ and that they exhibit different toxicological properties.^49^

A large number of analytical methods are described in the literature for the analysis of PFAS in various matrices,^52–56^ but there are only a few publications dealing with the quantification of PFAS in feed.^57–59^ In particular, the quantification of branched isomers can be challenging, as it requires an analytical method that separates the branched isomers not only from their linear isomers but also, ideally, from each other. Furthermore, differences in instrument sensitivity, optimal collision energy, and the predominant ion transitions among the various branched isomers can introduce quantification bias.^60^ In the case of PFOS, it has also been shown that the isomer profile of the analytical standard used may cause differences in the quantification.^61^ To assess the comparability of quantitative results for branched isomers, only a few proficiency tests (PTs) are regularly available for feed and food matrices. These PTs primarily focus on the branched isomers of PFOS and PFHxS, indicating a need for additional comparative studies on other branched isomers.

For this study, a feed material, hay grown on a PFAS-contaminated field in the region of Brilon-Scharfenberg, North Rhine-Westphalia, Germany, was utilized. The hay was analyzed as part of an interlaboratory comparison (ILC), using different extraction and purification methods. All laboratories used high-performance liquid chromatography coupled to tandem mass spectrometry (HPLC-MS/MS) or high-resolution MS for quantification and determined the content of linear PFAS in the sample. Three laboratories determined branched PFAS isomers in addition to the linear analytes. Key aims of this interlaboratory comparison were whether reliable analytical methods for PFAS quantification in feed are available, the comparison of sample preparation methods and the quantification of branched PFAS in a contaminated sample.

## 2 Material and Methods

All participants of the interlaboratory comparison used their own chemicals, standards, sample preparation methods, and instrumentation. The following chemicals, standards, sample preparation methods, and instruments were used by the organizing laboratory (laboratory 6).

### 2.1 Design of the Interlaboratory Comparison

This ILC was carried out to evaluate whether comparable results can be achieved by the participating laboratories regarding the analysis of PFAS in feed. In particular, the study focused on the short- and long-chain PFAAs, including their branched isomers, and was conducted from October 2021 to February 2022. For this purpose, a homogenized hay sample (30–60 g) from a contaminated test area in Brilon-Scharfenberg (Germany; North Rhine-Westphalia) was sent to all participating laboratories for analysis, without specifying a method. A summary of the methods used can be found in Section 2.4 and in the Supporting Information (SI) in Tables S12 and S13. A reporting form was provided, in which the participating laboratories entered their results for the levels of the individual EFSA-4 in μg/kg (based on 88% dry weight, dw), the sum of the EFSA-4, and their limits of quantification (LOQ). In addition, the content of the branched and linear perfluorocarboxylic acids C_4_–C_14_, and the perfluorosulfonic acids C_4_–C_10_, could be reported in μg/kg (based on 88% dw) along with their respective limits of quantification. Furthermore, general method information was requested, for example, accreditation according to DIN EN ISO/IEC 17025,^62^ sample weight, moisture content of the sample, use of isotopically labeled internal standards, use of recovery standards, use of a matrix calibration, sample preparation technique, extraction and purification methods, chromatographic separation, and detection methods. The received data were analyzed by the organizers.

### 2.2 Preparation of the Test Material

The hay sample was harvested from an approximately 10 ha large former arable area in Brilon-Scharfenberg in North Rhine-Westphalia, Germany, which had been treated with a compost mixture contaminated with PFAS through sewage sludge.^40^ After the environmental pollution was discovered in 2006, the arable land was under constant observation by the Regional Office for Nature, Environment and Consumer Protection North Rhine-Westphalia and has not been actively farmed ever since. As part of a monitoring program, the total PFAS depot of the polluted arable land was estimated at approximately 390 kg. In preparation for this study, naturally contaminated samples from seven subareas of the affected arable land were presampled. Previous analysis of the grass sample revealed PFAS of different chain lengths and their precursors.^40^

The hay (first cut) was harvested in May 2021 and pressed into hay bales. The hay bales were then transported to the German Federal Institute for Risk Assessment (BfR) and stored in a dry, cool, and dark place until analysis. For the PFAS analysis, samples were taken from all hay bales and from different locations within the same bale, respectively. A subsample of 1 kg hay was sent to the organizing laboratory.

The hay was milled to 0.5 mm with the ultracentrifugal mill (ZM 200, Retsch GmbH, Haan, Germany) and subsequently homogenized for 3 h in a drum hoop mixer (RF27DT80K4/IS, J. Engelsmann AG, Ludwigshafen am Rhein, Germany). For storage and shipment, 30–60 g of the sample were filled into polypropylene containers.

### 2.3 Chemicals

Acetonitrile and methanol for the sample preparation and HPLC-MS/MS measurements were purchased from Biosolve BV (Valkenswaard, Netherlands) in ULC/MS quality. Magnesium sulfate (4 g) and sodium chloride (1 g) were obtained as a ready-to-use mixture in an extraction salt package from Agilent Technologies (Waldbronn, Germany). Dispersive solid-phase extraction (dSPE) agents (150 mg primary secondary amine, 15 mg Envi Carb, 900 mg MgSO_4_) Supel QuE and the graphitized carbon black cartridge (Supelclean ENVI-Carb SPE Tube, 250 mg, 3 mL) were acquired from Supelco (Sigma-Aldrich Chemie GmbH, Taufkirchen, Germany). Ammonium acetate and formic acid (mass fraction *w* = 99%) were obtained from Biosolve in ULC/MS quality. Ammonia (*w* = 25%) and acetic acid (*w* = 100%) for analysis (EMSURE grade) were bought from Sigma-Aldrich Chemie GmbH. Ethylene glycol (*w* = 100%) was purchased in Analar Normapur quality from VWR International GmbH (Darmstadt, Germany). Fully deionized water, prepared by a water distiller (GFL 2001/2, GFL Gesellschaft für Labortechnik mbH, Burgwedel, Germany), has been used for this study.

All standards, ^13^C-labeled internal standards, and recovery standards of the perfluorocarboxylic acids and the perfluorosulfonic acids were purchased from Wellington Laboratories (Guelph, Canada) at a concentration of 50 μg mL^–1^ for the native compounds and 2 μg mL^–1^ for the ^13^C-labeled compounds in methanol. A detailed overview of all standards used for the quantification can be found in the Supporting Information (SI) in Tables S2–S5. In addition, the preparation steps of the stock solutions of the standards used can be found in the SI on page S3.

### 2.4 Instrumental Conditions

An Agilent Technologies 1290 Infinity II instrument was used as the HPLC system. To avoid possible cross-contamination from the system, all polytetrafluoroethylene (PTFE) parts were replaced or removed from the instrument. The chromatographic separation was achieved using an Agilent InfinityLab Poroshell 120 EC-C18 analytical column (2.1 × 150 mm, 2.7 μm particle size) at a flow rate of 0.25 mL min^–1^. A Phenomenex SecurityGuard Standard, C18 (2 mm × 4 mm), was used as the guard column, and an Agilent InfinityLab Poroshell 120 EC-C18 (3.0 mm × 50 mm, 2.7 μm particle size) served as the delay column. Mobile phase A consisted of water with 2 mmol/L ammonium acetate and 0.1% acetic acid, and mobile phase B of methanol/acetonitrile 60:40 by volume. The following gradient conditions were applied: 20% B at 0.5 min, 55% B at 2 min, 80% B at 10 min, 98% B at 13 min, 98% B at 17.5 min, and 20% B at 18.5 min.

The HPLC was coupled to a 6495B MS/MS fitted with an electrospray ionization interface operating in negative mode. Nitrogen gas (5.0 from Linde plc, Dublin, Ireland) served as nebulizer gas with a pressure of 35 psi at 120 °C. The sheath gas had a temperature of 300 °C and a flow rate of 10 mL/min. The capillary voltage was kept at 3 kV, and the mass spectrometer was operated in dynamic multiple reaction mode (d-MRM) with the resolution setting unit to unit. Collision energies, cell acceleration voltage, and the respective transitions are shown in Tables S6 and S7 of the SI

The confirmation of the PFBA and PFPeA levels was achieved by using a Restek Raptor PolarX analytical column (2.1 × 50 mm, 2.7 μm particle size) at a flow rate of 0.5 mL min^–1^. The chromatographic separation is based on a Hydrophilic Interaction Liquid Chromatography (HILIC) mechanism. Mobile phase A consisted of water with 10 mmol/L ammonium formate and 0.05% acetic acid, and mobile phase B consisted of methanol/acetonitrile (60:40 by volume) with 0.05% acetic acid. The following gradient conditions were applied: 95% B at the start, 65% B at 4.5 min, and 95% B at 6 min. MassHunter for QQQ Version 10.0 was used for data acquisition and quantification.

### 2.5 Homogeneity and Stability Test

To determine the homogeneity, ten 0.3 g aliquots of the milled and homogenized hay sample were analyzed using a QuEChERS (Quick, Easy, Cheap, Effective, Rugged and Safe) method. In addition, 0.3 g samples from each container sent to the participating laboratories were also analyzed to determine the homogeneity. The stability test of the PFAS analytes in the sample was conducted simultaneously by applying storage conditions at room temperature in high-density polyethylene (HDPE) containers under the exclusion of air over a period of 3 months.

For analysis, 0.3 g of the sample was weighed into a 50 mL centrifuge tube together with 100 μL of the internal standard stock solution II (SI Table S3), followed by 10 mL of distilled water, 10 mL of acetonitrile, and 150 μL of formic acid. The sample was then shaken for 5 min and placed in the ultrasonic bath (Elma S40H, Papenburg, Germany) for another 5 min. 4 g of MgSO_4_ and 1 g of NaCl were added as salting-out sorbents. The sample was shaken on a Multi Reax (Heidolph Scientific Products GmbH, Schwabach, Germany) for 1 min and centrifuged at 2330 rcf (relative centrifugal force) for 15 min (Multifuge 1 S R, Heraeus, Hanau, Germany). The acetonitrile phase, containing the analytes, was transferred to a 15 mL centrifuge tube. The dSPE agents were added for cleanup. The centrifuge tube was then shaken on the Multi Reax for 5 min and centrifuged at 2330 rcf for 15 min. The supernatant was removed and subjected to further purification using a graphitized carbon black cartridge. The cartridge was equilibrated sequentially with 4 mL of 0.1% ammonia in acetonitrile, followed by 4 mL of pure acetonitrile. The precleaned sample extract was then loaded onto the cartridge, and the eluate was collected in a 15 mL centrifuge tube. Subsequently, the cartridge was eluted twice with 1.5 mL of 0.1% ammonia in acetonitrile, and the resulting eluate was pooled in the same centrifuge tube. The combined eluate was then evaporated to dryness at 50 °C under a gentle nitrogen stream. To prevent sample loss during evaporation, 10 μL of ethylene glycol was added as a keeper. This step is omitted in a newer version of the method due to contamination of the measurement instrument, which manifested as the buildup of a viscous film at the hexabore capillary outlet on the back of the desolvation assembly, a component of the analyzer. Finally, 440 μL of a 1% formic acid solution in a 2:1 methanol/water mixture and 50 μL of the recovery standard (stock solution II) were added to the dried sample. The sample was vortexed thoroughly and transferred to a polypropylene vial for analysis. The measurement on the HPLC-MS/MS was carried out as described in Section 2.4. To confirm PFBA and PFPeA levels in accordance with the EURL POPs Guidance Document, the samples were diluted 1:50 with methanol by volume and analyzed on a HILIC column, which employs a different separation mechanism compared to the C18 column.

### 2.6 Information on Participating Laboratories

The nine participating laboratories from Germany used their own house methods for sample preparation and quantification without receiving specific instructions. This approach allowed full flexibility and nevertheless led to several analytic methods with comparable performance. Six of the nine laboratories used analytical methods accredited according to DIN EN ISO/IEC 17025 (no accreditation of the method: laboratories 6, 8, and 9).^62^ All laboratories used ^13^C-labeled internal standards for quantification of PFAS. This has the advantage that any analyte losses during sample preparation or intensity fluctuations during the measurement can be compensated. Isotopically labeled standards are not commercially available for all investigated linear and branched PFAS, so that PFAS with a similar chain length or, in the case of the branched isomers, the respective linear isomer had to be used for the quantification. Five out of nine laboratories reported that they used isotopically labeled standards to determine the recovery of the internal standards. The recovery standard consists of a mixture of isotopically labeled PFAS compounds, different from those used as internal standards. The recovery standard is added to the final measurement sample. This approach allows for the assessment of the recovery efficiency of the internal standards, providing an additional layer of quality control for the sample preparation without influencing the quantification results. An overview of the achieved recoveries of the organizing laboratory is shown in Table S8 of the SI and ranged between 62 and 103% for the EFSA-4.

Extraction techniques included solid–liquid extraction with methanol (three laboratories) or acetonitrile (four laboratories) and ion-pair extraction with methyl *tert*-butyl ether and *tert*-butyl amine (two laboratories). Three of the four laboratories using acetonitrile also added buffer salts during extraction, following the commonly described QuEChERS approach. One laboratory used 0.1% ammonia in acetonitrile for the extraction. Five laboratories used solid-phase extraction with a weak anion exchange sorbent for the sample cleanup. A QuEChERS purification step with dSPE reagents was performed by two laboratories. One of those laboratories added a further SPE purification step with graphitized carbon black. The two laboratories using the ion-pair extraction did not further purify the extract. The impact of these differences on the results is further described in Section 3.5. All participants used HPLC techniques for the analysis. Only one laboratory used high-resolution MS as a detector, the other participants analyzed the test sample with low-resolution MS/MS instruments. A detailed overview on the reported methods of the participating laboratories can be found in Table S12 of the SI.

### 2.7 Statistical Evaluation

Nine laboratories submitted results for the requested analytical scope. For PFPeS, PFTrDA, and PFTeDA (abbreviations: Table 1), fewer than the minimum required number of seven laboratories submitted results. Specifically, results for PFPeA were reported by three laboratories, while results for PFTrDA and PFTeDA were reported by five laboratories each. Therefore, the statistical parameters for the analyses were calculated for information only and were marked accordingly. A pre-evaluation of the data was carried out to identify outlying laboratories. The identification criterion was a systematic deviation of the laboratory from the other laboratories. Assigned values (consensus value of participants) were only calculated for PFAS if more than 2/3 of the results were above the LOQ and less than 1/3 of all results were outside the range of ±50% of the median. Results outside the range of ±50% of the median were defined as outliers and were excluded from the calculation of the assigned values. Further, to minimize the effects of potential outliers on the assigned value as well as on precision data, the evaluation was carried out by robust statistics according to ISO 13528.^59,63^

Assigned value and reproducibility standard deviation were evaluated with the algorithm A described in ISO 13528.^63^ This method of robust statistics is also referred to as the Huber estimator;^64^ it was calculated using the *hubers* function of the MASS package^65^ in R.^66^

For the calculation of *z*-scores, a target standard deviation (σ_ILC_) of 20% was defined, which is in compliance with the criteria for trueness according to the European Union Reference Laboratory for halogenated POPs in Feed and Food (EURL POPs) guidance document and ISO 13528.^63^

The *z*-score for the assessment of laboratory performance was calculated according to

1 $$z=\frac{(x_{i}-x_{\mathrm{ILC}})}{\sigma_{\mathrm{ILC}}}$$

with *x*_i_ as the measurement result reported by the participant and *x*_ILC_ as the assigned value.

### 2.8 Comparison of MRM Transitions for the Quantification of Branched PFAS

In order to compare MRM transitions of selected branched PFAS (br-PFOS, br-PFOA, br-PFDS, and br-PFNS), the test material of the ILC was quantified using the linear isomer but with different mass transitions according to the procedure described above. The transition for the respective internal standard was adjusted as well.

## 3 Results and Discussion

### 3.1 Homogeneity and Stability of the Test Sample

The homogeneity and stability of the sample were determined according to ISO 13528.^63^ For this purpose, 20 aliquots of the hay were analyzed over a three-month period, with two sets of 10 samples analyzed at different times. The analyte is considered sufficiently homogeneously distributed if at least the extended condition for homogeneity according to ISO 13528, point B.2.3, is fulfilled.^63^ Applying this condition, all analytes were found to be sufficiently homogeneous and stable (Tables S9 and S10, SI). The levels of PFBA and PFPeA in the hay sample were confirmed by measuring 10 samples with a HILIC column. Details of the results are shown in Table S11 of the SI.

### 3.2 PFAS Content in the Hay Sample

The PFAS median content in the hay sample ranged between 0.11 μg/kg based on 88% dw for PFPeS and 148 μg/kg based on 88% dw for PFBA (Table 1 and SI Table S12). The highest contributions to the sum of the EFSA-4 are those of PFOS and PFOA. But also, PFAS that are not part of the EFSA-4 group contribute considerably, with 80% including the branched isomers (referring to the median content) to the total PFAS content in the hay (SI Table S12). These numbers are of quite concern as they show that the contamination of the field led to an uptake of PFAS by the plants. Even though hay is a feed plant, food crops could behave similarly, as summarized in a recent review from Lesmeister et al.^67^

Another exposure route for humans is the transfer of the PFAS from the feed to farm animals that generate food. There are currently no regulations, such as maximum levels of PFAS in feed, in the European Union. However, in a recent opinion by our institute, reference values for PFOS were set to 0.07–0.42 μg/kg dw depending on the intended livestock.^68^ These values are exceeded more than one hundred-fold in the analyzed hay sample (Table 1).

In a study on the transfer of PFAS from feed to dairy cows using hay collected from the same contaminated area in Germany as in this study, the milk and muscle tissue showed high contamination levels of PFOS, PFOA, PFHxS, and PFBS.^69^ A study on PFAS concentrations in eggs after feeding chickens with contaminated feed concluded that PFAS are transferred from the hen into the eggs, which were highly contaminated.^57^ Both studies used feed that was even more contaminated than the hay in this study. A study conducted on pigs investigating the ingestion of PFAS from feed, contaminated in the same order of magnitude as the hay in this study, showed that the concentration attained in the meat reaches levels above the regulatory limits (PFOS: 0.30 μg/kg; PFOA: 0.80 μg/kg; PFNA: 0.20 μg/kg; and PFHxS: 0.20 μg/kg).^70,71^ In summary, the transfer of PFAS from contaminated hay to food is a realistic threat that could endanger consumers, an opinion also held by EFSA.^48^

### 3.3 Branched PFAS

PFAS were produced using telomerization or electrochemical fluorination. While telomerization only produces linear isomers, electrochemical fluorination results in a variety of cyclic and branched isomers as byproducts.^14,49,50^ The quantification of branched PFAS is challenging due to the lack of standards and limited chromatographic separation. In addition, there is a need for further method harmonization. Hence, only three of the nine laboratories provided data on branched PFAS other than br-PFOS (Table 2). Because of the lack of standard chemicals, the quantification was performed using the linear isomer of the respective PFAS. According to Version 2.0 of the EURL POPs Guidance Document on PFAS, br-PFOS can be quantified using the linear isomer, based on the average concentration from the two mass transitions.^59^ This approach may also be applicable to other branched PFAS.

**Table 2:** Reported Content of Branched PFAS^a^

PFAS	lab 6	lab 8	lab 9	median	*σ*_rel_ [%]
**br-PFOS**	14.43	10.00	8.80	10.00	27
**br-PFOA**	12.14	11.02	11.00	11.00	6
**br-PFNA**	0.10	0.19	—	0.14	44
**br-PFHxS**	0.37	—	—	—	—
**br-PFBS**	—	—	—	—	—
**br-PFPeS**	—	—	—	—	—
**br-PFHpS**	—	1.60	—	1.60	—
**br-PFNS**	154.14	87.10	100.00	100.00	31
**br-PFDS**	103.79	76.50	86.00	86.00	16
**br-PFBA**	—	—	—	—	—
**br-PFPeA**	0.26	1.11	—	0.69	88
**br-PFHxA**	1.62	0.98	0.75	1.00	40
**br-PFHpA**	0.45	0.41	0.86	0.45	44
**br-PFDA**	0.05	0.07	—	0.06	26
**br-PFUnDA**	0.06	0.15	—	0.10	60
**br-PFDoDA**	0.74	1.07	—	0.91	26
**br-PFTrDA**	1.32	0.86	1.00	1.00	22
**br-PFTeDA**	0.86	—	1.20	1.00	23
**Extraction**	Acetonitrile^b^	Methanol	Ion pair	—	—
**Cleanup**	Modified QuEChERS	SPE	—	—	—

a Content reported as μg/kg based on 88% dry weight with extraction and cleanup method . The content refers to the sum of the branched isomers.

b QuEChERS approach using buffer salts.

The hay sample analyzed in this study clearly showed that branched PFAS can significantly contribute to the total PFAS content of feed. Br-PFNS and br-PFDS displayed the highest concentration of all the branched PFAS (Figure 1 and Table 2). This case shows that the branched isomers can be the dominant species in a given test sample.

**Figure 1:**
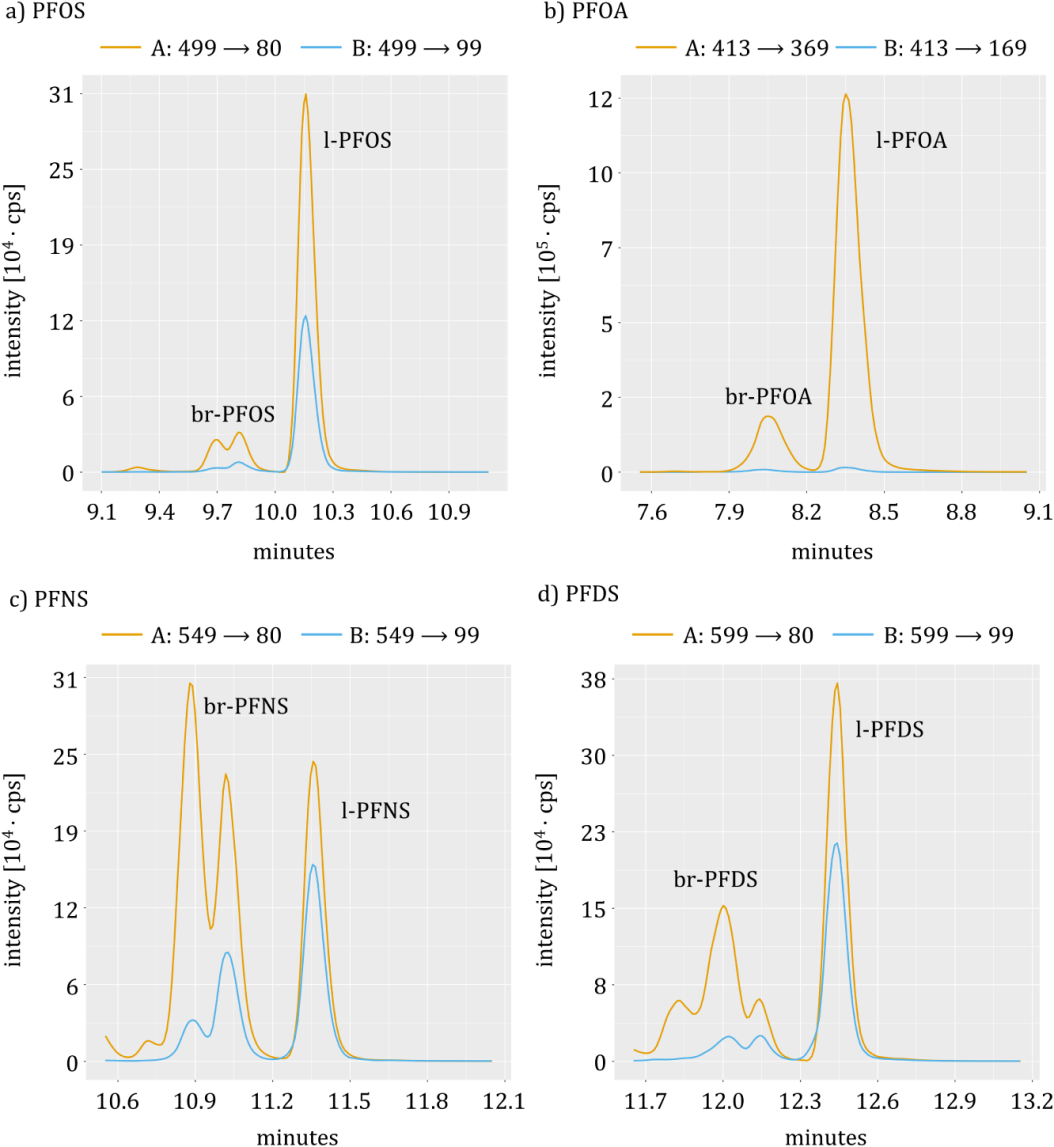
Comparison of the hay sample chromatograms measured by the organizing laboratory. Two multi-reaction mode (MRM) transitions of branched PFAS are shown. Comparison of two transitions, A and B (Table 3), for (a) PFOS, (b) PFOA, (c) PFNS, and (d) PFDS.

Due to the challenges stated above, the quantification of branched isomers, except for the isomers of PFOS, is not standard procedure at the moment. The relative standard deviation of the reported concentration for the branched PFAS ranges between 6% and 60% (Table 2). The EURL POPs recommended a within-laboratory precision of maximum 25% for monitoring purposes (for those substances that do not fall under current regulation).^59^ More than half of the branched PFAS, where more than one laboratory reported a value, are within these limits. Based on these results, future studies with more participants and a higher availability of appropriate standards might lead to a more comprehensive data set, from which more conclusions can then be drawn. The choice of MRM transition influences the quantitative results of branched PFAS (Table 3 and Figure 1). Depending on the mass transition, the chromatogram of the isomer peaks can change, which can lead to different quantitative results. The impact of the MRM transition is high, with values differing up to 77%. Branched PFAS are commonly quantified using the respective linear isomer as calibration and internal standard. However, matrix effects can influence the response of the linear isomers differently compared to the branched isomers, and these effects may vary depending on the extraction method as well as on the efficiency of the cleanup procedure. Consequently, the results highlight the need for appropriate reference standards specifically designed for accurate quantification of branched PFAS isomers.

**Table 3:** Comparison of MRM Transitions Used for Quantification^a^

analyte	transition	q1 *m*/*z*	q3 *m*/*z*	μg/kg 88% dw	average ± σ
	MRM transition				
br-PFOS	A	499	80	15	18 ± 4.5
	B	499	99	22	
br-PFOA	A	413	369	12	9.1 ± 3.5
	B	413	169	6.6	
br-PFNS	A	549	80	149	95 ± 77
	B	549	99	40	
br-PFDS	A	599	80	103	63 ± 58
	B	599	99	21	

a Comparison of two transitions, A and B (Figure 1), with *m*/*z* value of the parent ion (q1)­and the daughter ion (q3). Result reported as μg/kg based on 88% dry weight (dw)­and average of both transitions with standard deviation (σ). MRM: multi-reaction mode.

### 3.4 Evaluation of Reported Limits of Quantification

The reported limits of quantification (LOQs) range between 0.01 μg/kg and 10 μg/kg based on 88% dw (Figure 2 and SI, Table S13). Most reported LOQs are in the range of 0.1 μg/kg based on 88% dw. By evaluating potential correlations between the reported LOQs and the analytical instruments used, it was observed that the laboratory employing high-resolution mass spectrometry (HRMS) reported the highest LOQs for the EFSA-4 PFAS (0.50 μg/kg, based on 88% dry weight). Considering that only one laboratory used HRMS, and that the achievable LOQs are influenced by various method-specific parameters, such as the extraction procedure and cleanup step, no conclusive evidence for instrument-related differences could be established.

**Figure 2:**
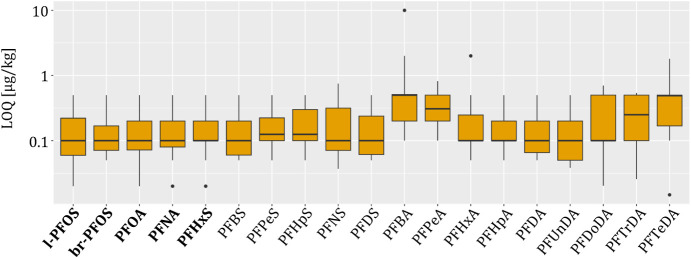
Limits of quantification (LOQ) range of the PFAS (88% dw). Logarithmic scale. The box shows the interquartile range, that is, the middle 50% of scores or the range between the 25th and 75th percentiles. The line in the middle of the box represents the median value. The upper and lower whiskers represent the highest and lowest values within the 1.5-times interquartile range in the positive and negative directions, respectively. Values outside of this range are represented as dots. The EFSA-4 PFAS are highlighted in bold.

At the moment, there are no regulations in place for PFAS in feed or foods of plant origin in the European Union.^71^ Considering the reference values for PFOS (laying hen: 0.42 μg/kg dw; cattle: 0.14 μg/kg; sheep: 0.21 μg/kg dw; and fattening pigs: 0.07 μg/kg dw) in complete feed set in an opinion by our institute, most participating laboratories would be able to quantify the amounts set by the reference values.^68^ The low values set for fattening pigs’ feed (PFOS: 0.07 μg/kg dw; PFOA: 0.05 μg/kg dw; PFNA: n.a.; and PFHxS: 0.06 μg/kg dw) present a challenge to various laboratories. This could indicate that improvements in the methods for quantification of certain PFAS in feed might be needed.

The LOQs are all in a similar range; only PFBA, PFPeA, and the longer-chain perfluorocarboxylic acids PFDoDA, PFTrDA, and PFTeDA exhibit slightly higher LOQs based on the median value. A higher variability in the LOQs was also observed for those analytes (Figure 2). For PFBA and PFPeA, only one specific MRM transition is available, and their retention on a standard reversed-phase C18 column is lower compared to the other PFAS included in this study. Consequently, these analytes are particularly prone to coelution and matrix effects, which were also observed in this matrix. For the longer-chain perfluorocarboxylic acids PFDoDA, PFTrDA, and PFTeDA, the higher variability in the LOQs may be attributed to adsorption on plastic containers or other materials used during sample preparation. It has been described in the literature that adsorption onto different container materials depends on factors such as solvent composition, contact time, and temperature. Adsorption generally occurs and increases with the chain length of the PFCA.^72^

### 3.5 Comparison of Sample Preparation Methods

Different sample preparation and analysis methods were used by the nine laboratories (Tables S12 and S13, SI). Eight out of nine used HPLC-MS/MS, whereas the remaining laboratory used high-resolution MS. Since only one laboratory used a different MS technique, a statistical comparison is not appropriate.

Extraction was carried out either by solid–liquid extraction with methanol (three laboratories) or acetonitrile (four laboratories) or ion-pair extraction with methyl *tert*-butyl ether and *tert*-butyl amine (two laboratories). Three out of the four laboratories using acetonitrile performed the extraction according to the QuEChERS approach with buffer salts. The remaining laboratory extracted with 0.1% ammonia in acetonitrile. The EFSA-4 content of 103 μg/kg, based on 88% dry weight, which is outside the range, does not originate from the laboratory applying the non-QuEChERS approach (Table 1). Therefore, and due to the small data set, no distinction was made within the acetonitrile group between extractions performed with or without buffer salts when evaluating possible effects on the sum of the EFSA-4. The different extraction solvents did not influence the content of the sum of EFSA-4 (Figure 3). Although the modified QuEChERS cleanup yielded the highest EFSA-4 median concentration, this result may not be representative due to the limited number of participating laboratories. Considering the result of 172 μg/kg based on 88% dw of the SPE group, it becomes evident that the use of SPE or a modified QuEChERS method as a cleanup step has no significant influence on the determination of the sum of the EFSA-4 concentration (Figure 3).

**Figure 3:**
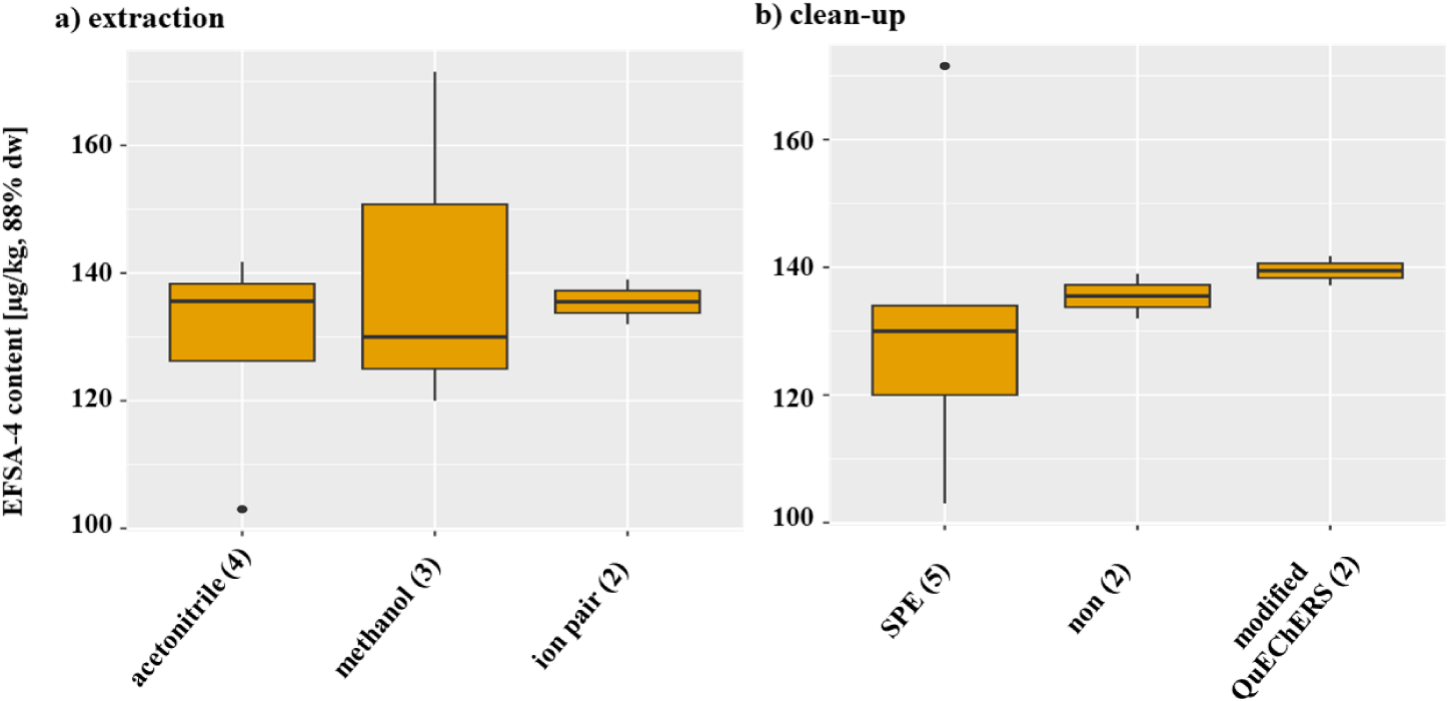
Comparison of (a) extraction solvents and (b) cleanup method. (a) Content (88% dw) of the sum of EFSA-4 after extraction either with acetonitrile, methanol or ion pair extraction (ion pair), depicted as boxplots. Numbers in parentheses represent the number of laboratories that used this extraction solvent or method. (b) Content (μg/kg based on 88% dry weight, dw) of the sum of EFSA-4 after a cleanup either with SPE, modified QuEChERS, or no cleanup, depicted as boxplots. The box shows the interquartile range, that is, the middle 50% of scores or the range between the 25th and 75th percentiles. The line in the middle of the box represents the median value. The upper and lower whiskers represent the highest and lowest values within the 1.5-times interquartile range in the positive and negative directions, respectively. Values outside of this range are represented as dots.

The comparisons described in this study are based on relatively small sample sizes and should be reevaluated in a designated experiment or an ILC with more participants per method.

### 3.6 Laboratory Performances

The performance of the participating laboratories is expressed as *z*-score. Absolute *z*-scores under two are rated as acceptable performance, absolute values between two and three are rated as questionable, and *z*-scores above three are deemed unsatisfactory.

The relative reproducibility standard deviation (RSD_R_) can be used as a criterion for fitness for purpose (ISO 13528). For the individual analytes, it ranged from 11.1% for l-PFOS to 47.4% for PFNS, and was 17.6% for total PFOS and 10.0% for the sum of EFSA-4.

For nine analytes, the RSD_R_ of the reported results was in agreement with the target standard deviation (20%); for the other nine analytes, the RSD_R_ was higher than 20% (Table 4).

**Table 4:** Assigned Values in μg/kg Based on 88% Dry Weight (Dw), Robust Standard Deviation in μg/kg; 88% (Dw) and Relative Standard Deviation (RSD_R_)­^a^

PFAS	assigned value	robust standard deviation (*s*_R_)	relative standard deviation (RSD_R_)
	μg/kg; 88% dw	μg/kg; 88% dw	%
l-PFOS	61.82	6.87	11.1
br-PFOS	11.13	3.05	27.4
total-PFOS	72.35	12.74	17.6
PFNA	0.29	0.08	28.6
PFOA	60.01	9.40	15.7
PFHxS	1.26	0.18	14.1
sum EFSA-4	133.42	13.37	10.0
PFBS	0.54	0.07	12.1
PFPeS	—	—	—
PFHpS	0.27	0.09	32.4
PFNS	—	—	—
PFDS	101.21	35.32	34.9
PFBA	149.10	27.19	18.2
PFPeA	40.65	11.00	27.1
PFHxA	9.90	1.40	14.2
PFHpA	2.89	0.42	14.6
PFDA	0.48	0.11	22.2
PFUnDA	0.31	0.08	25.6
PFDoDA	0.58	0.14	23.3
PFTrDA	—	—	—
PFTeDA	—	—	—

a Outliers were removed .

Overall, the *z*-scores lie mostly between −1 and +1, and 92% fall in the range between −2 and +2 (143 single results), underlining the quality of the analytical methods used in this ILC (Figure 4 and SI, Table S13). An absolute *z*-score ≤2 is deemed acceptable by the EURL POPs for proficiency tests in feed.^73^ PFPeS, PFTrDA, and PFTeDA were excluded from the *z*-score evaluation because less than a third of laboratories provided a value above the LOQ. PFNS was excluded because more than a third of all provided values were outside of the interquartile range. The observed variability in the reported PFNS concentrations may be attributed to a potential coelution of the branched and linear isomers, depending on the chromatographic conditions applied by the laboratories. With a median content of 100 μg/kg based on 88% dw for the branched isomers and 47 μg/kg based on 88% dw for the linear isomer, PFNS shows the highest proportion of branched isomers compared to its linear form in the hay sample (Tables 1 and 2).

**Figure 4:**
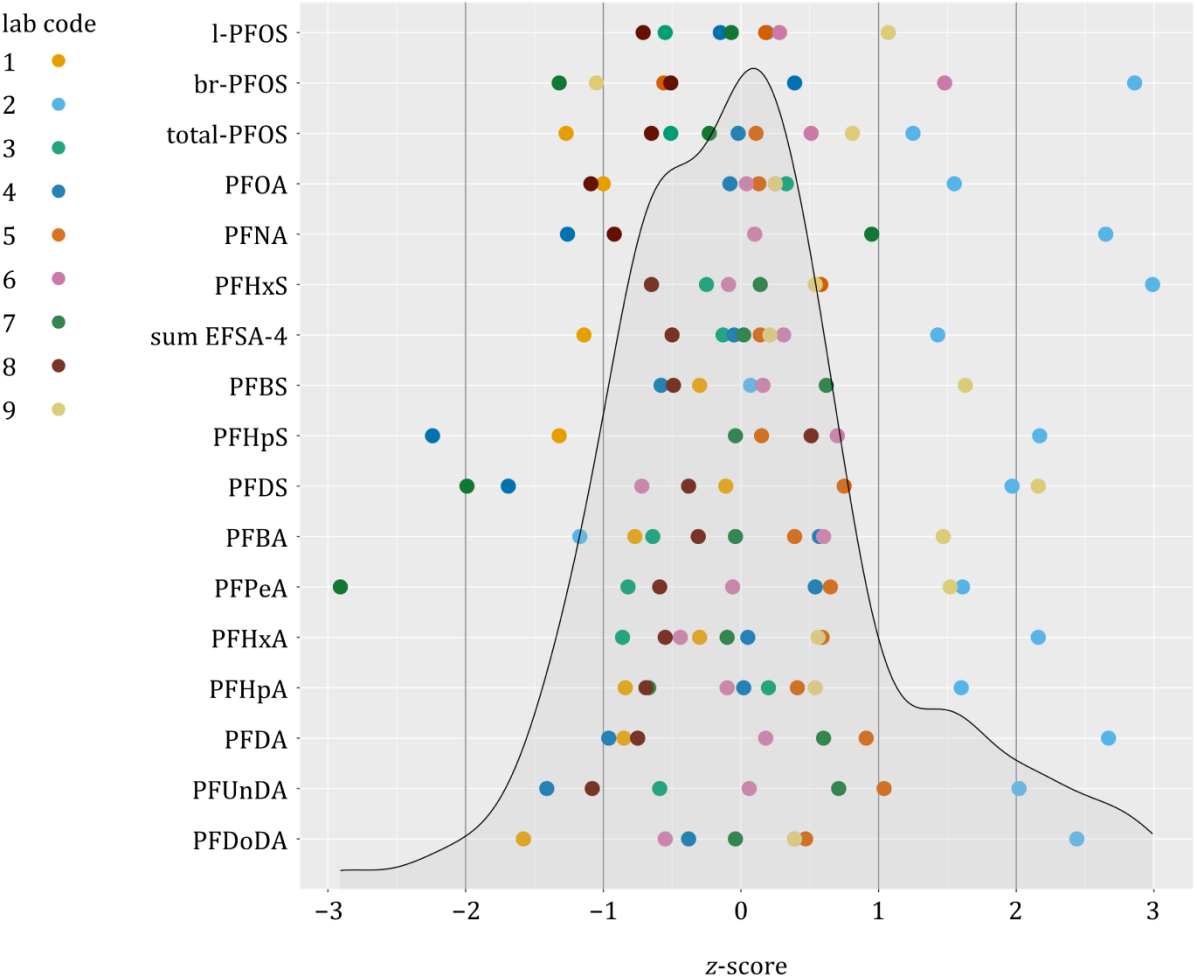
*z* **-**Scores. PFPeS, PFTrDA, and PFTeDA were excluded because less than a third of laboratories provided values above the LOQ. PFNS was excluded because more than a third of all provided values were outside the interquartile range.

All laboratories using nonaccredited methods (6, 8, and 9) achieved *z*-scores below 2 (with the exception of laboratory 9 for PFDS). There is no difference in the quality of the results between accredited and nonaccredited methods. Laboratory 2 showed a relatively high value for PFHxS. This could indicate a coelution with br-PFHxS, which was only quantified by laboratory 6 (0.37 μg/kg; 88% dw). However, laboratory 2 showed relatively high absolute *z*-scores (Figure 4 and SI, Table S13); thus, it might also be a more general issue with the analytical method used by this particular laboratory. Laboratory 3 used a matrix-matched calibration, which resulted in excellent *z*-scores. With an average absolute *z*-score of 0.4 and none higher than 0.86, laboratory 3 performed best of all laboratories. Regardless of the impracticality of this approach for routine analysis, due to the small number of participating laboratories, no clear conclusions can be drawn regarding the effect of a matrix-matched calibration on analytical performance.

The statistical evaluation showed that the quantification of PFAS in the hay sample yielded to comparable results, even when using different extraction and cleanup techniques. Nevertheless, the study was based on a small data set from nine laboratories. These laboratories applied three different extraction methods and two different cleanup techniques, which may have introduced potential bias into the interpretation of the findings. A more detailed comparative study on the hay sample itself, or another feed material with a broader range of analytical results, particularly emphasizing the influence of extraction and cleanup methodologies, would be required to identify potential correlations between these aspects.

Further, the results of the study show that there is a need for improvement and harmonization of analytical methods quantifying branched PFAS, which could be used for monitoring purposes and for studies on the toxicokinetics in agricultural livestock and humans. These data could serve as a basis for the establishment of management measures, like maximum levels in feed, especially when considering the transfer to edible animal parts such as eggs, milk, or meat. Efforts should be made to improve the availability of branched native and isotopically labeled standards. Furthermore, the availability of additional proficiency tests or comparative studies that include branched isomers other than br-PFOS and br-PFHxS in feed, but also food, would help to better assess the accuracy on the quantification of branched isomers. Crops cultivated on contaminated arable land, such as that in Brilon-Scharfenberg, North Rhine-Westphalia, Germany, which was the origin of the hay sample used in this study, could provide suitable plant-based feed or food matrices for future comparative studies.

## Supplementary Material



## Notes

Additional information from the organizing laboratory on standards, MRM transitions, recoveries of the internal standards, and the test of homogeneity. Overview of the assigned values, achieved LOQs, and z-scores. Further information on analytical methods (e.g., sample intake, extraction, cleanup) from the participating laboratories (PDF)

## Abbreviations

Table 1: Abbreviations for the individual poly- and perfluoroalkyl substances can be found in Table 1
BfR: German Federal Institute for Risk Assessment
dSPE: dispersive solid-phase extraction
dw: dry weight
HILIC: hydrophilic interaction liquid chromatography
HDPE: high-density polyethylene
QuEChERS: quick, easy, cheap, effective, rugged, and safe
ECHA: European Chemicals Agency
EFSA: European Food Safety Authority
EFSA-4: perfluorooctanesulfonic acid, perfluorooctanoic acid, perfluorononanoic acid, and perfluorohexanesulfonic acid
EURL POPs: European Union Reference Laboratory for halogenated Persistent Organic Pollutants in Feed and Food
HPLC: high-performance liquid chromatography
ILC: interlaboratory comparison
LOQ: limit of quantification
MRM: multiple reaction monitoring
MS: mass spectrometry
MS/MS: tandem mass spectrometry
PFAAs: perfluoroalkyl acids
PFAS: poly- and perfluoroalkyl substances
POPs: persistent organic pollutants
PSA: primary secondary amine
SI: Supporting Information
SPE: solid-phase extraction
σ: standard deviation
σrel: relative standard deviation
