# Supplementary material for: A Broad Mixture of Linear and Branched Perfluoroalkyl Substances (PFAS) in Hay: Results of an Interlaboratory Comparison
Source: J Agric Food Chem. 2026 Feb 9;74(6):5769–80. doi: 10.1021/acs.jafc.5c09362 (PMC12921859; doi:10.1021/acs.jafc.5c09362)
Supplement: Supplementary file 1 [file jf5c09362_si_001.pdf]

Supporting Information

A broad mixture of linear and branched perfluoroalkyl substances (PFAS) in hay — results of an interlaboratory comparison

Anne Jahnke[a§*], Konstantin Simon[a§], Caroline Goedecke-Röber[a], Janine Kowalczyk[a], Michael Weiss[a] Anja Lüth[a]

[a] German Federal Institute for Risk Assessment, Department Safety in the Food Chain, Max-Dohrn-Str. 8-10, 10589 Berlin, Germany

[*] Email: anne.jahnke@bfr.bund.de

[§] A.J and K.S contributed equally to this work

Table of Content

Abbreviation S1
Information on the preparation of the analytical standards from the organizing laboratory S3
Concentration of used standard solutions from the organizing laboratory S4
Instrumental parameters from the organizing laboratory S8
Recoveries of the internal standards S10
Test for homogeneity of the hay sample S11
Confirmation of PFBA and PFPeA content using a HILIC column S13
Method information of the participating laboratories S14
Results of the interlaboratory study S16

List of Tables

Overview of the used abbreviations and their meaning S2
Stock solutions of native PFASs S4
Internal standards S5
13C recovery standards S6
Calibration levels S7
General mass spectrometric parameters S8
MRM transitions S8
Recoveries of the internal standards of ten hay sample replicates S10
Results of the homogeneity test for PFBA - PFHpS S11
Results of the homogeneity test for PFOS - PFTeDA S12
Comparisons of the PFBA and PFPeA content of the organizing laboratory S13
Overview of the method information from laboratory 1 - 5 .... S14
Overview of the method information from laboratory 6 - 9 .... S14
Reported results of the participants and assigned values S16
Reported limits of quantification (LOQ) from the participating laboratories S17
Calculated z-scores S18

Abbreviation

Table S1. Overview of the used abbreviations and their meaning

Abbreviation	Meaning
**NA**	non-applicable (missing value)
**σ**	standard deviation
**lb**	lower bound
**R-**	recovery standard
**σrel**	relative standard deviation
**HILIC**	Hydrophilic Interaction Liquid Chromatography
**PFAS**	poly- and perfluoroalkyl substances
**EFSA**	European Food Safety Authority
**QuEChERS**	Quick, Easy, Cheap, Effective, Rugged and Safe
**l-PFOS**	linear perfluorooctanesulfonic acid
**br-PFOS**	branched perfluorooctanesulfonic acid
**t-PFOS**	total perfluorooctanesulfonic acid (sum of l-PFOS and br-PFOS)
**PFOA**	perfluorooctanoic acid
**PFNA**	perfluorononanoic acid
**PFHxS**	perfluorohexanesulfonic acid
**total EFSA-4 (lower bound)**	sum of t-PFOS, PFOA, PFNA, PFHxS
**PFBS**	perfluorobutanesulfonic acid
**PFPeS**	perfluoropentanesulfonic acid
**PFHpS**	perfluoroheptanesulfonic acid
**PFNS**	perfluorononanesulfonic acid
**PFDS**	perfluorodecanesulfonic acid
**PFBA**	perfluorobutanoic acid
**PFPeA**	perfluoropentanoic acid
**PFHxA**	perfluorohexanoic acid
**PFHpA**	perfluoroheptanoic acid
**PFDA**	perfluorodecanoic acid
**PFUnDA**	perfluoroundecanoic acid
**PFDoDA**	perfluorododecanoic acid
**PFTrDA**	perfluorotridecanoic acid
**PFTeDA**	perfluorotetradecanoic acid
**br-PFOA**	branched perfluorooctanoic acid
**br-PFNA**	branched perfluorononanoic acid
**br-PFHxS**	branched perfluorohexanesulfonic acid
**br-PFHpS**	branched perfluoroheptanesulfonic acid
**br-PFNS**	branched perfluorononanesulfonic acid
**br-PFDS**	branched perfluorodecanesulfonic acid
**br-PFPeA**	branched perfluoropentanoic acid
**br-PFHxA**	branched perfluorohexanoic acid
**br-PFHpA**	branched perfluoroheptanoic acid
**br-PFDA**	branched perfluorodecanoic acid
**br-PFUnDA**	branched perfluoroundecanoic acid
**br-PFDoDA**	branched perfluorododecanoic acid
**br-PFTrDA**	branched perfluorotridecanoic acid
**br-PFTeDA**	branched perfluorotetradecanoic acid

Information on the preparation of the analytical standards from the organizing laboratory

Stock solution I was prepared by diluting and mixing the native compound solutions with methanol to achieve a final concentration of 1 µg mL^{-1}. Stock solutions II–V were each prepared by diluting the previous stock solutions 1:10 with methanol.

The internal standard stock solution I was prepared by diluting the purchased ^{13}C standards with methanol to a concentration of 100 ng mL^{-1}. This solution was further diluted with methanol to receive internal standard stock solution II with a final concentration of 10 ng mL^{-1}. The solutions for the recovery standard were prepared in the same way as the internal standard. The stock solutions were stored at -20 °C in the dark in polypropylene tubes. From these stock solutions, calibration levels in the range from 0.01 ng/mL to 80 ng/mL were prepared.

Concentration of used standard solutions from the organizing laboratory

Table S2. Stock solutions of native PFASs

			stock solutions				
		single standards	I	II	III	IV	V
native PFASs	abbreviation	[ng/mL]	[ng/mL]	[ng/mL]	[ng/mL]	[ng/mL]	[ng/mL]
Perfluoro-n-butanoic acid	PFBA	50000	1000	100	10	1	0.1
Perfluoro-n-pentanoic acid	PFPeA	50000	1000	100	10	1	0.1
Perfluoro-n-hexanoic acid	PFHxA	50000	1000	100	10	1	0.1
Perfluoro-n-heptanoic acid	PFHpA	50000	1000	100	10	1	0.1
Perfluoro-n-octanoic acid	PFOA	50000	1000	100	10	1	0.1
Perfluoro-n-nonanoic acid	PFNA	50000	1000	100	10	1	0.1
Perfluoro-n-decanoic acid	PFDA	50000	1000	100	10	1	0.1
Perfluoro-n-undecanoic acid	PFUnDA	50000	1000	100	10	1	0.1
Perfluoro-n-dodecanoic acid	PFDoDA	50000	1000	100	10	1	0.1
Perfluoro-n-tridecanoic acid	PFTrDA	50000	1000	100	10	1	0.1
Perfluoro-n-tetradecanoic acid	PFTeDA	50000	1000	100	10	1	0.1
Perfluoro-n-hexadecanoic acid	PFHxDA	50000	1000	100	10	1	0.1
Perfluoro-n-octadecanoic acid	PFODA	50000	1000	100	10	1	0.1
Potassium perfluoro-1-butanesulfonate	PFBS	44200	884	88.4	8.84	0.884	0.0884
Sodium perfluoro-1-pentanesulfonate	PFPeS	46900	938	93.8	9.38	0.938	0.0938
Sodium perfluoro-1-hexanesulfonate	PFHxS	47300	946	94.6	9.46	0.946	0.0946
Sodium perfluoro-1-heptanesulfonate	PFHpS	47600	952	95.2	9.52	0.952	0.0952
Sodium perfluoro-1-octanesulfonate	PFOS	47800	956	95.6	9.56	0.956	0.0956
Sodium perfluoro-1-nonanesulfonate	PFNS	48000	960	96	9.6	0.96	0.096
Sodium perfluoro-1-decanesulfonate	PFDS	48200	964	96.4	9.64	0.964	0.0964
Sodium perfluoro-1-dodecanesulfonate	PFDoDS	48400	968	96.8	9.68	0.968	0.0968

Table S3. Internal standards

mass-labeled PFAS extraction standards		internal standard I [ng/mL]	internal standard II *used for calibration levels* [ng/mL]	internal standard III *used for samples* [ng/mL]
Perfluoro-n-[13C4]butanoic acid	MPFBA	2000	100	10
Perfluoro-n-[13C5]pentanoic acid	M5PFPeA	2000	100	10
Perfluoro-n-[1.2.3.4.6-13C5]hexanoic acid	M5PFHxA	2000	100	10
Perfluoro-n-[1.2.3.4-13C4]heptanoic acid	M4PFHpA	2000	100	10
Perfluoro-n-[13C8]octanoic acid	M8PFOA	2000	100	10
Perfluoro-n-[13C9]nonanoic acid	M9PFNA	2000	100	10
Perfluoro-n-[1.2.3.4.5.6-13C6]decanoic acid	M6PFDA	2000	100	10
Perfluoro-n-[1.2.3.4.5.6.7-13C7]undecanoic acid	M7PFUdA	2000	100	10
Perfluoro-n-[1.2-13C2]dodecanoic acid	MPFDoA	2000	100	10
Perfluoro-n-[1.2-13C2]tetradecanoic acid	M2PFTeDA	2000	100	10
Sodium perfluoro-1-[2.3.4-13C3]butanesulfonate	M3PFBS	1858.56	92.93	9.293
Sodium perfluoro-1-[1.2.3-13C3]hexanesulfonate	M3PFHxS	1891.83	94.59	9.459
Sodium perfluoro-1-[13C8]octanesulfonate	M8PFOS	1913.25	95.66	9.566

Table S4. ^{13}C recovery standards

mass-labeled PFAS recovery standards		recovery standard I [ng/mL]	recovery standard II *used for calibration levels* [ng/mL]	recovery standard III *used for samples* [ng/mL]
Perfluoro-n-[2.3.4-13C3]butanoic acid	M3PFBA	2000	100	10
Perfluoro-n-[1.2-13C2]octanoic acid	M2PFOA	2000	100	10
Perfluoro-n-[1.2-13C2]decanoic acid	MPFDA	2000	100	10
Sodium perfluoro-1-[1.2.3.4-13C4]octanesulfonate	MPFOS	1912.60	95.63	9.56

Table S5. Calibration levels

	**level I [ng/mL]**	**level II [ng/mL]**	**level III [ng/mL]**	**level IV [ng/mL]**	**level V [ng/mL]**	**level VI [ng/mL]**	**level VII [ng/mL]**	**level VIII [ng/mL]**	**level IX [ng/mL]**	**level X [ng/mL]**	**level XI [ng/mL]**	**level XII [ng/mL]**
PFBA	0.01	0.025	0.05	0.1	0.5	1	2.5	5	10	20	40	80
PFPeA	0.01	0.025	0.05	0.1	0.5	1	2.5	5	10	20	40	80
PFHxA	0.01	0.025	0.05	0.1	0.5	1	2.5	5	10	20	40	80
PFHpA	0.01	0.025	0.05	0.1	0.5	1	2.5	5	10	20	40	80
PFOA	0.01	0.025	0.05	0.1	0.5	1	2.5	5	10	20	40	80
PFNA	0.01	0.025	0.05	0.1	0.5	1	2.5	5	10	20	40	80
PFDA	0.01	0.025	0.05	0.1	0.5	1	2.5	5	10	20	40	80
PFUnDA	0.01	0.025	0.05	0.1	0.5	1	2.5	5	10	20	40	80
PFDoDA	0.01	0.025	0.05	0.1	0.5	1	2.5	5	10	20	40	80
PFTrDA	0.01	0.025	0.05	0.1	0.5	1	2.5	5	10	20	40	80
PFTeDA	0.01	0.025	0.05	0.1	0.5	1	2.5	5	10	20	40	80
PFHxDA	0.01	0.025	0.05	0.1	0.5	1	2.5	5	10	20	40	80
PFODA	0.01	0.025	0.05	0.1	0.5	1	2.5	5	10	20	40	80
PFBS	0.00884	0.0221	0.0442	0.0884	0.442	0.884	2.21	4.42	8.84	17.68	35.36	70.72
PFPeS	0.00938	0.02345	0.0469	0.0938	0.469	0.938	2.345	4.69	9.38	18.76	37.52	75.04
PFHxS	0.00946	0.02365	0.0473	0.0946	0.473	0.946	2.365	4.73	9.46	18.92	37.84	75.68
PFHpS	0.00952	0.0238	0.0476	0.0952	0.476	0.952	2.38	4.76	9.52	19.04	38.08	76.16
PFOS	0.00956	0.0239	0.0478	0.0956	0.478	0.956	2.39	4.78	9.56	19.12	38.24	76.48
PFNS	0.0096	0.024	0.048	0.096	0.48	0.96	2.40	4.80	9.6	19.20	38.40	76.80
PFDS	0.00964	0.0241	0.0482	0.0964	0.482	0.964	2.41	4.82	9.64	19.28	38.56	77.12
PFDoDS	0.00968	0.0242	0.0484	0.0968	0.484	0.968	2.42	4.84	9.68	19.36	38.72	77.44

Each calibration level contains 10 ng/mL of the internal and recovery standard.

Instrumental parameters from the organizing laboratory

Table S6. General mass spectrometric parameters

Parameters MS		Parameters Ion Source	
resolution	unit to unit	gas temp.	120 °C
fragmentor voltage	380 V	gas flow	11 L/min
polarity	negative	nebulizer preasure	35 psi
quadrupol temperatures	100 °C	sheath gas temp.	300 °C
		sheath gas flow	10 L/min

Table S7. MRM transitions

Compound Name	Precursor Ion	Product Ion	Collision Energy (V)	Cell Accelerator Voltage (V)	Retention Time (min)
13C-PFBA	217	217	0	1	4.5
13C-PFBA	217	172	4	1	4.5
13C-PFDA	519	473.9	6	1	11
13C-PFDA	519	223	16	1	11
13C-PFDoDA	615	569.9	8	1	13
13C-PFDoDA	615	320	19	1	13
13C-PFHpA	367	321.9	7	1	7.2
13C-PFHpA	367	172	17	1	7.2
13C-PFHxA	318	273	4	4	6.1
13C-PFHxA	318	121	20	1	6.1
13C-PFNA	472	426.9	6	1	9.6
13C-PFNA	472	223	16	1	9.6
13C-PFOA	421	375.9	7	1	8.3
13C-PFOA	421	172	16	1	8.3
13C-PFPeA	268	268	0	1	5.2
13C-PFPeA	268	223	4	1	5.2
13C-PFTeDA	715	670	12	1	14.5
13C-PFTeDA	715	270	25	6	14.5
13C-PFUnDA	570	525	8	1	12.2
13C-PFUnDA	570	274	19	1	12.2
PFBA	213	213	0	1	4.5
PFBA	213	169	4	1	4.5
PFDA	513	469	8	1	11
PFDA	513	219	16	1	11
PFDoDA	613	569	9	1	13
PFDoDA	613	319	17	1	13
PFHpA	363	319	8	1	7.2
PFHpA	363	169	16	1	7.2
PFHxA	313	269	4	4	6.1
PFHxA	313	119	20	1	6.1

Compound Name	Precursor Ion	Product Ion	Collision Energy (V)	Cell Accelerator Voltage (V)	Retention Time (min)
PFHxDA	813	769	14	1	15.3
PFHxDA	813	169	34	4	15.3
PFNA	463	419	8	1	9.6
PFNA	463	219	16	1	9.6
PFOA	413	369	8	1	8.3
PFOA	413	169	4	1	8.3
PFOdA	913	869	16	1	15.9
PFOdA	913	169	40	1	15.9
PFPeA	263	263	0	1	5.2
PFPeA	263	219	4	1	5.2
PFTeDA	713	669	12	1	14.5
PFTeDA	713	169	28	6	14.5
PFTrDA	663	619	12	1	13.8
PFTrDA	663	169	25	4	13.8
PFUnDA	562.9	519	7	1	12.2
PFUnDA	562.9	269	17	1	12.2
R-PFBA	216	216	0	1	4.5
R-PFBA	216	172	4	1	4.5
R-PFDA	515	470	8	1	11
R-PFDA	515	220	17	1	11
R-PFOA	415	369.9	8	1	8.3
R-PFOA	415	170	16	1	8.3
13C-PFBS	302	99	32	1	5.6
13C-PFBS	302	80	50	1	5.6
13C-PFHxS	402	99	38	1	7.6
13C-PFHxS	402	80	50	1	7.6
13C-PFOS	507	99	56	1	10.1
13C-PFOS	507	80	56	1	10.1
PFBS	299	99	32	2	5.6
PFBS	299	80	52	1	5.6
PFDoS	699	99	58	3	14
PFDoS	699	80	62	1	14
PFDS	599	99	52	1	12.4
PFDS	599	80	64	1	12.4
PFHpS	449	99	40	1	8.6
PFHpS	449	80	48	1	8.6
PFHxS	399	99	36	1	7.6
PFHxS	399	80	48	2	7.6
PFNS	549	99	52	1	11.3
PFNS	549	80	76	2	11.3
PFOS	499	99	40	1	10.1
PFOS	499	80	56	2	10.1
PFPeS	349	99	44	1	6.6
PFPeS	349	80	48	1	6.6
R-PFOS	503	99	46	1	10.1
R-PFOS	503	80	56	1	10.1

Recoveries of the internal standards

Table S8. Recoveries of the internal standards of ten hay sample replicates

	Recoveries of the internal standards [%]												
**Sample**	**^{13}C-PFBA**	**^{13}C-PFPeA**	**^{13}C-PFBS**	**^{13}C-PFHxA**	**^{13}C-PFHpA**	**^{13}C-PFHxS**	**^{13}C-PFOA**	**^{13}C-PFNA**	**^{13}C-PFOS**	**^{13}C-PFDA**	**^{13}C-PFUnDA**	**^{13}C-PFDoDA**	**^{13}C-PFTeDA**
hay 1	70	155	286	70	67	64	78	94	76	72	78	69	75
hay 2	75	164	329	70	71	66	82	99	77	76	80	74	78
hay 3	73	170	318	69	70	67	81	97	78	77	80	72	77
hay 4	72	152	294	67	67	62	78	97	72	72	75	68	72
hay 5	72	160	310	69	68	66	82	97	78	75	78	72	76
hay 6	71	143	281	69	68	65	80	96	75	72	76	72	76
hay 7	75	167	330	69	70	64	81	98	77	75	79	73	77
hay 8	78	174	342	68	70	65	83	101	76	77	80	73	76
hay 9	75	163	308	71	72	67	86	100	81	77	81	73	80
hay 10	75	163	308	74	74	70	84	103	81	79	84	78	79

Test for homogeneity and stability of the hay sample

Table S9. Results of the homogeneity test for PFBA - PFHpS

		content µg/kg; 88% dw								
sample	replicate	PFBA	PFPeA	PFBS	PFHxA	PFPeS	PFHpA	PFHxS	PFOA	PFHpS
hay 1	1	169.92	40.7	0.58	9.39	0.12	2.94	1.27	57.9	0.31
hay 1	2	158.69	38.8	0.56	9.04	0.11	2.57	1.26	50.66	0.33
hay 2	1	175.41	42.2	0.56	9.11	0.13	2.78	1.23	58.91	0.28
hay 2	2	143.67	36.34	0.51	8.89	0.12	2.58	1.11	49.76	0.34
hay 3	1	161.89	37.44	0.55	8.71	0.12	2.67	1.18	54.67	0.29
hay 3	2	152.82	37.24	0.57	9.25	0.12	2.52	1.21	50.8	0.33
hay 4	1	170.64	41.77	0.56	9.92	0.11	3.03	1.15	57.73	0.32
hay 4	2	147.67	38.37	0.52	8.75	0.12	2.39	1.26	51.21	0.34
hay 5	1	166.32	39.04	0.57	8.75	0.11	2.78	1.23	54.95	0.29
hay 5	2	153.45	38.23	0.56	9.06	0.11	2.51	1.25	50.02	0.35
hay 6	1	167.09	40.69	0.56	9.01	0.11	2.75	1.23	68.91	0.33
hay 6	2	146.39	38.27	0.54	8.74	0.10	2.48	1.13	50.46	0.33
hay 7	1	165.92	40.84	0.55	9.17	0.10	2.86	1.28	59.76	0.30
hay 7	2	156.74	37.08	0.55	9.74	0.11	2.67	1.31	51.41	0.34
hay 8	1	157.40	37.95	0.53	10.51	0.12	3.52	1.35	72.13	0.33
hay 8	2	151.01	38.94	0.54	8.67	0.11	2.41	1.25	50.74	0.34
hay 9	1	170.23	40.73	0.60	9.13	0.12	2.87	1.19	60.69	0.30
hay 9	2	146.55	37.62	0.53	9.07	0.13	2.64	1.23	51.87	0.35
hay 10	1	164.72	40.22	0.55	8.88	0.11	2.82	1.27	59.09	0.30
hay 10	2	147.73	36.22	0.55	10.2	0.12	2.93	1.14	52.08	0.33
		**Cochran's C-test**								
C		0.304	0.354	0.408	0.454	0.21	0.589	0.247	0.377	0.237
Ccrit 95		0.602	0.602	0.602	0.602	0.602	0.602	0.602	0.602	0.602
Ccrit 99		0.717	0.717	0.717	0.717	0.717	0.717	0.717	0.717	0.717
C<Ccrit		yes	yes	yes	yes	yes	yes	yes	yes	yes
		**Homogeneity test**								
General average		158.7139	38.935	0.5515	9.1995	0.1158	2.736	1.2255	55.6872	0.3215
Standard deviation of sample averages s_x		2.8424	0.8037	0.0134	0.2661	0.008	0.1155	0.0467	2.8602	0.0091
Within-sample standard deviation s_w		12.8694	2.1999	0.023	0.6119	0.0068	0.3233	0.0569	7.7868	0.0259
Between-sample standard deviation s_s		0	0	0	0	0.0064	0	0.0238	0	0
Standard deviation for proficiency assessment σ_{ILC}		31.7428	7.787	0.1103	1.8399	0.0232	0.5472	0.2451	11.1374	0.0643
s_s / σ_{ILC}		0	0	0	0	0.2779	0	0.097	0	0
**Test for homogeneity ($s_s \leq \sigma_{ILC}$)**		**passed**	**passed**	**passed**	**passed**	**passed**	**passed**	**passed**	**passed**	**passed**

Table S10. Results of the homogeneity test for PFOS - PFTeDA

		**content µg/kg; 88% dw**									
sample	replicate	br-PFOS	PFOS	PFDA	PFNS	PFUdA	PFDS	PFDoA	PFTrDA	PFDoDS	PFTeDA
hay 1	1	13.94	65.00	0.49	49.00	0.32	87.86	0.51	0.46	11.42	0.28
hay 1	2	11.25	60.28	0.48	49.10	0.30	87.75	0.47	0.33	12.10	0.23
hay 2	1	14.05	65.51	0.52	49.58	0.32	91.14	0.51	0.44	11.70	0.25
hay 2	2	10.91	59.34	0.48	47.25	0.29	96.81	0.50	0.34	12.98	0.25
hay 3	1	13.68	62.22	0.49	46.46	0.28	81.8	0.48	0.4	11.01	0.24
hay 3	2	11.03	58.66	0.48	49.13	0.31	97.04	0.51	0.35	14.58	0.26
hay 4	1	15.62	69.17	0.52	50.31	0.32	92.41	0.54	0.44	11.21	0.26
hay 4	2	11.36	60.56	0.47	48.75	0.30	91.45	0.47	0.32	12.18	0.23
hay 5	1	13.74	61.19	0.49	44.46	0.30	83.04	0.52	0.43	9.04	0.25
hay 5	2	13.73	69.49	0.51	54.75	0.33	97.27	0.49	0.36	13.46	0.23
hay 6	1	17.35	69.02	0.50	47.18	0.30	86.41	0.51	0.41	11.46	0.25
hay 6	2	11.30	62.22	0.48	48.42	0.30	92.02	0.47	0.33	12.39	0.21
hay 7	1	14.44	64.86	0.50	47.24	0.31	86.39	0.52	0.41	12.71	0.25
hay 7	2	11.37	60.00	0.46	47.76	0.29	89.77	0.48	0.33	12.12	0.20
hay 8	1	15.31	65.09	0.49	47.03	0.31	87.60	0.52	0.45	11.50	0.26
hay 8	2	11.14	58.99	0.47	47.91	0.28	86.80	0.46	0.32	12.36	0.21
hay 9	1	14.96	67.98	0.53	47.95	0.31	85.73	0.54	0.46	10.82	0.24
hay 9	2	11.30	59.47	0.47	47.26	0.30	86.96	0.45	0.32	12.43	0.21
hay 10	1	14.10	63.10	0.49	45.53	0.31	84.44	0.51	0.46	11.04	0.28
hay 10	2	11.44	60.47	0.48	48.16	0.31	91.29	0.49	0.36	12.34	0.22
		**Cochran's C-test**									
C		0.29	0.184	0.264	0.809	0.213	0.415	0.333	0.176	0.471	0.213
Ccrit 95		0.602	0.602	0.602	0.602	0.602	0.602	0.602	0.602	0.602	0.602
Ccrit 99		0.717	0.717	0.717	0.717	0.717	0.717	0.717	0.717	0.717	0.717
C<Ccrit		yes	yes	yes	no	yes	yes	yes	yes	yes	yes
		**Homogeneity test**									
General average		13.1016	63.13	0.4894	48.1626	0.3038	89.1989	0.4986	0.3868	11.9414	0.241
Standard deviation of sample averages s_x		0.6151	1.6952	0.0081	0.9446	0.0064	2.3175	0.0072	0.0132	0.4523	0.0114
Within-sample standard deviation s_w		2.5121	4.4854	0.0242	2.5579	0.0154	5.2901	0.0349	0.0736	1.4418	0.0281
Between-sample standard deviation s_s		0	0	0	0	0	0	0	0	0	0
Standard deviation for proficiency assessment σ_{ILC}		2.6203	12.626	0.0979	9.6325	0.0608	17.8398	0.0997	0.0774	2.3883	0.0482
s_s / σ_{ILC}		0	0	0	0	0	0	0	0	0	0
**Test for homogeneity ($s_s \leq \sigma_{ILC}$)**		**passed**	**passed**	**passed**	**passed**	**passed**	**passed**	**passed**	**passed**	**passed**	**passed**

Confirmation of PFBA and PFPeA content using a HILIC column

Table S11. Comparisons of the PFBA and PFPeA content of the organizing laboratory

	content µg/kg 88% dw					
	C18 column		HILIC column		deviation HILIC results %	
**Sample**	**PFBA**	**PFPeA**	**PFBA**	**PFPeA**	**PFBA**	**PFPeA**
hay 1	158.7	38.8	150.7	39.4	-5.1	1.6
hay 2	143.7	36.3	145.8	38.8	1.5	6.8
hay 3	152.8	37.2	144.9	39.3	-5.2	5.7
hay 4	147.7	38.4	148.0	40.2	0.2	4.7
hay 5	153.5	38.2	151.3	39.8	-1.4	4.0
hay 6	146.4	38.3	147.1	40.1	0.5	4.8
hay 7	156.7	37.1	152.1	40.6	-3.0	9.6
hay 8	151.0	38.9	149.3	38.7	-1.1	-0.7
hay 9	146.5	37.6	140.4	38.3	-4.2	1.8
hay 10	147.7	36.2	147.0	40.4	-0.5	11.6

Method information of the participating laboratories

Table S12. Overview of the method information as reported from laboratory 1 - 5

method information from participating laboratories (lab 1 - 5)					
	**lab 1**	**lab 2**	**lab 3**	**lab 4**	**lab 5**
**method accreditation**	yes	yes	yes	yes	yes
**sample intake**	1 g	1 g	2 g	2 g	0.75 g and 1.5 g
**use of internal standards**	yes	yes	yes	yes	yes
**extraction method**	• 10 mL water + 10 mL acetonitrile<br>• addition of internal standard<br>• 1 min shaking and 60 min sonication<br>• addition of buffer-salt mixture<br>• shaking and 5 min centrifugation	• addition of internal standard<br>• 20 mL methanol<br>• 15 min shaking and 60 min sonication<br>• in between shaking by hand every 15 min<br>• 15 min centrifugation	• 3x with methanol	• alkaline extraction with methyl tert-butyl ether (MTBE) using tetrabutylammonium hydrogen sulfate (TBA) as ion pair reagent<br>• concentration of the ether phase	• modified QuEChERS method<br>• extraction with acetonitrile
**cleanup**	• 1:1 dilution of a 50% (5 mL) aliquot with water<br>• SPE (WAX) clean-up with a 80% (8 mL) aliquot<br>**Conditioning:**<br>• 2 mL of formic acid water (0.1% formic acid)<br>• 2 mL of methanol<br>• apply 2 mL of water / extract<br>**Sample Loading**<br>• apply 8 mL water / extract<br>**Washing:**<br>• 2 mL of formic acid water (0.1% formic acid)<br>• 2 mL of methanol<br>**Elution:**<br>1.8 mL with 0.1% ammonia in methanol<br>**Post-SPE treatment:**<br>• Evaporate to dryness<br>• Reconstitute in 1 mL (final volume) of a 1:2 mixture of 0.1% formic acid / methanol	• SPE (Strata-X-AW. 33 µm Polymeric Weak Anion. 60 mg/3 mL)<br>**Conditioning:**<br>• 2 mL of 1% formic acid<br>• 2 mL of methanol<br>• 2 mL of water<br>**Sample Loading:**<br>• 1:1 dilution of supernatant with water<br>**Elution:**<br>• Fraction 1: 2 mL of 0.1% formic acid<br>• Fraction 2: 2 mL of methanol<br>• Fraction 3: 2 mL of 0.1% ammonium hydroxide in methanol<br>**Post-SPE treatment:**<br>• addition of 5 µL glycerol to fraction 3<br>• Evaporate to dryness<br>• Reconstitute in the initial mobile phase conditions: 500 µL (20 mM ammonium acetate solution: MeOH. 90:10 v/v).	• SPE Phenomenex -x-aw 33; 100 mg. 6 mL	• no cleanup	• dSPE
**instrument**	LC-MS/MS	LC-MS/MS	LC-MS/MS	LC-MS/MS	LC-MS/MS
**chromatographic column**	Zorbax Eclipse XDB-C18. 1.8-Micron. 4.6 x 100mm	C18. Gemini® 3 µm C18 110Å. 150 x 2 mm	no information	reverse phase C18 column	reverse phase C18 column
**eluent**	**A:** 0.1 mmol ammonium acetate in 0.1% formic acid<br>**B:** 0.1 mmol ammonium acetate in methanol	**A:** 20 mM ammonium acetate<br>**B:** methanol	no information	no information	Methanol, acetonitrile and a weak ammonium acetate buffer

Table S13. Overview of the method information as reported from laboratory 6 - 9

method information from participating laboratories (lab 6 - 9)				
	**lab 6**	**lab 7**	**lab 8**	**lab 9**
**method accreditation**	no	yes	no	no
**sample intake**	0.3 g	0.05 - 2 g	0.5 g	0.5 g
**use of internal standards**	yes	yes	yes	yes
**extraction method**	• addition of internal standard<br>•10 mL water + 10 mL acetonitrile + 150 µL formic acid<br>• 5 min shaking and 5 min sonication<br>• addition of buffer-salt mixture<br>• 1 min shaking and 15 min centrifugation	• extraction with 0.1 % NH_3 in acetonitrile	• according to DIN 38414-14:2011-08<br>• 10 mL methanol with internal standard<br>• 60 min sonication at 45°C<br>• in between regularly shaking<br>• allow the solid components to settle overnight	• ion pair extraction with methyl tert-butyl ether (MTBE) and tetrabutylammonium hydrogen sulfate (TBA) in the presents of a puffer at pH 10<br>• concentration and resuspension in methanol
**cleanup**	• dSPE (150 mg primary secondary amine. 15 mg Envi Carb. 900 mg MgSO4)<br>• 5 min shaking<br>• 15 min centrifugation<br>• SPE clean-up with graphitized carbon black<br>**Conditioning:**<br>• 4 mL of 0.1% ammonia in acetonitrile<br>• 4 mL of acetonitrile<br>**Sample Loading:**<br>• precleaned acetonitrile supernatant<br>**Elution:**<br>• direct collection of the eluate + 2x 1.5 mL 0.1% ammonia in acetonitrile<br>**Post-SPE treatment:**<br>• addition of 10 µL glycerol<br>•evaporation to dryness under a gentle stream of nitrogen at 50 °C<br>• reconstitution in 440 µL 1% formic acid solution in a 2:1 methanol/water mixture and addition of the recovery standard	• Envi-Carb SPE und StrataX SPE	• according to DIN 38414-14:2011-08<br>• SPE: weak anion exchange sorbent on a polymer basis (Macherey-Nagel HR-XAW. 60 mg/3 mL)<br>**Conditioning:**<br>• 2 mL of 0.1% formic acid in methanol<br>• 2 mL of methanol<br>• 2 mL of water<br>**Sample Loading:**<br>• A mixture of 1 mL of 0.1% formic acid in water and 1 mL of the methanolic sample extract was applied to the cartridge<br>**Washing:**<br>• 2 mL water<br>• 2 ml acetone/acetonitrile/formic acid, 50/50/1<br>• 2 mL methanol<br>**Elution:**<br>• 3 mL 0.1% ammonia in methanol<br>**Post-SPE treatment:**<br>•evaporation to dryness under a gentle stream of nitrogen at 40 °C<br>• reconstitution in 1 mL of a water–methanol mixture corresponding to the initial LC mobile phase conditions.	• no clean-up
**instrument**	LC-MS/MS	LC-MS/MS	LC-MS/MS	HRMS (Orbitrap)
**chromatographic column**	• Poroshell 120 EC-C18 column (2.1 × 150 mm. 2.7 µm)	XBrigde BEH 150 x 2.1 mm 1.8 µm	Zorbax Eclipse Plus C18 (50 mm x 2.1 mm. 1.9 µm)<br>- Eluent A 10 mM Ammo	C18-UPLC column
**eluent**	**A:** 2 mmol/l ammonium acetate and 0.1% acetic acid<br>**B:** methanol/acetonitrile 60:40	**A:** 0.1 mmol/l ammonium acetate and 5% acetonitrile<br>**B:** methanol/acetonitrile 40:60	**A:** 10 mmol/l ammonium acetate<br>**B:** 10 mmol/l ammonium acetate in methanol	**A:** 2 mmol/l ammonium acetate in water/methanol 95/5<br>**B:** 2 mmol/l ammonium acetate in methanol

Results of the interlaboratory study

Table S14. Reported results of the participants and assigned values including all linear and branched isomers. The total PFAS median content was calculated by the organizing laboratory.

	lab code (results in µg/kg; 88% dw)													
**analyte**	**1**	**2**	**3**	**4**	**5**	**6**	**7**	**8**	**9**	**mean**	**median**	**σ**	**σrel [%]**	**assigned value**
**l-PFOS**	NA	64.13	55.00	60.00	64.03	65.31	61.00	53.00	75.00	62.18	62.52	6.80	10.93	61.82
**br-PFOS**[a]	NA	17.48	10.00	12.00	9.89	14.43	8.20	10.00	8.80	11.35	10.00	3.16	27.80	11.13
**total-PFOS**	54.00	90.47[b]	65.00	72.00	73.92	80.00	69.00	63.00	84.00	72.38	72.00	11.26	15.56	72.35
**PFOA**	48.00	78.59	64.00	59.00	61.55	60.47	63.00	46.98	63.00	60.51	61.55	9.32	15.40	60.01
**PFNA**	0.22	0.45	NA	0.22	0.30	0.30	0.35	0.24	NA	0.30	0.30	0.08	27.81	0.29
**PFHxS**	1.10	2.02	1.20	1.10	1.41	1.24	1.30	1.10	1.40	1.32	1.24	0.29	21.97	1.26
**sum EFSA-4 (lb)**	103.00	171.53	130.00	132.00	137.18[d]	141.76	134.00	120.00	139.00[d]	134.27	134.00	18.30	13.63	133.42
**PFBS**	0.51	0.55	0.48	0.48	0.56	0.56	0.61	0.49	0.72	0.55	0.55	0.08	14.01	0.54
**PFPeS**	0.11	NA	NA	0.10	0.11	0.12	NA	NA	NA	0.11	0.11	0.01	6.48	NA
**PFHpS**	0.20	0.39	NA	0.15	0.28	0.31	0.27	0.30	NA	0.27	0.28	0.08	28.60	0.27
**PFNS**	44.00	74.05	NA	23.00	49.08	47.48	NA	42.90	90.00	52.93	47.48	22.14	41.83	NA
**PFDS**	99.00	141.04	NA	67.00	116.43	86.68	61.00	93.50	145.00	101.21	96.25	31.16	30.79	101.21
**PFBA**	126.00	114.23	130.00	166.00	160.84	166.96	148.00	140.00	193.00	149.45	148.00	24.66	16.50	149.10
**PFPeA**	34.00	53.72	34.00	45.00	45.94	40.16	17.00	35.89	53.00	39.86	40.16	11.38	28.54	40.65
**PFHxA**	9.30	14.18	8.20	10.00	11.06	9.02	9.70	8.82	11.00	10.14	9.70	1.79	17.64	9.90
**PFHpA**	2.40	3.81	3.00	2.90	3.12	2.83	2.50	2.49	3.20	2.92	2.90	0.44	15.11	2.89
**PFDA**	0.40	0.74	0.41	0.39	0.57	0.50	0.54	0.41	NA	0.49	0.46	0.12	24.40	0.48
**PFUnDA**	0.27	0.43	0.27	0.22	0.37	0.31	0.35	0.24	NA	0.31	0.29	0.07	23.02	0.31
**PFDoDA**	0.40	0.87	NA	0.54	0.64	0.52	0.58	0.63	NA	0.60	0.58	0.14	24.21	0.58
**PFTrDA**	0.20	1.09	NA	0.28	0.29	0.44	NA	0.24	NA	0.42	0.29	0.34	79.46	NA
**PFTeDA**	NA	0.92	NA	0.12	0.24	0.21	0.29	NA	NA	0.36	0.24	0.32	90.02	NA
**br-PFOA**[a]	NA	NA	NA	NA	NA	12.00	NA	11.00	11.00	11.33	11.00	0.58	5.09	NA
**br-PFNA**[a]	NA	NA	NA	NA	NA	0.10	NA	0.19	NA	0.15	0.15	0.06	43.89	NA
**br-PFHxS**[a]	NA	NA	NA	NA	NA	0.37	NA	NA	NA	0.37	0.37	NA	NA	NA
**br-PFHpS**[a]	NA	NA	NA	NA	NA	NA	NA	1.60	NA	1.60	1.60	NA	NA	NA
**br-PFNS**[a]	NA	NA	NA	NA	NA	154.00	NA	87.00	100.00	113.67	100.00	35.53	31.26	NA
**br-PFDS**[a]	NA	NA	NA	NA	NA	104.00	NA	77.00	86.00	89.00	86.00	13.75	15.45	NA
**br-PFPeA**[a]	NA	NA	NA	NA	NA	0.26	NA	1.11	NA	0.69	0.69	0.60	87.74	NA
**br-PFHxA**[a]	NA	NA	NA	NA	NA	1.62	NA	0.98	0.75	1.12	0.98	0.45	40.37	NA
**br-PFHpA**[a]	NA	NA	NA	NA	NA	0.45	NA	0.41	0.86	0.57	0.45	0.25	43.44	NA
**br-PFDA**[a]	NA	NA	NA	NA	NA	0.05	NA	0.07	NA	0.06	0.06	0.01	23.57	NA
**br-PFUnDA**[a]	NA	NA	NA	NA	NA	0.06	NA	0.15	NA	0.11	0.11	0.06	60.61	NA
**br-PFDoDA**[a]	NA	NA	NA	NA	NA	0.74	NA	1.07	NA	0.91	0.91	0.23	25.78	NA
**br-PFTrDA**[a]	NA	NA	NA	NA	NA	1.32	NA	0.86	1.00	1.06	1.00	0.24	22.24	NA
**br-PFTeDA**[a]	NA	NA	NA	NA	NA	0.86	NA	NA	1.20	1.03	1.03	0.24	23.34	NA
**total PFAS**											687.22			
**extraction**	acetonitrile[c]	methanol	methanol	ion pair	acetonitrile[c]	acetonitrile[c]	acetonitrile	acetonitrile	ion pair					
**cleanup**	SPE	SPE	SPE	/	modified QuEChERS	modified QuEChERS	SPE	SPE	/					

a Sum of all branched isomers
b The laboratory used a different method for the quantification of the total PFOS content.
c QuEChERS approach using buffer salts
d Linear PFOS used for the sum parameter

Table S15. Reported limits of quantification (LOQ) from the participating laboratories

	LOQ (µg/kg; 88% dw)												
	lab code												
**analyte**	**1**	**2**	**3**	**4**	**5**	**6**	**7**	**8**	**9**	**mean**	**median**	**σ**	**σrel [%]**
**l-PFOS**	NA	0.20	0.10	0.05	0.30	0.06	0.02	0.10	0.50	0.17	0.10	0.16	97
**br-PFOS**	NA	0.20	0.10	0.05	NA	0.06	NA	0.10	0.50	0.17	0.10	0.17	101
**PFOA**	0.10	0.20	0.08	0.05	0.25	0.10	0.02	0.10	0.50	0.16	0.10	0.15	95
**PFNA**	0.10	0.20	0.12	0.05	0.30	0.07	0.02	0.10	0.50	0.16	0.10	0.15	94
**PFHxS**	0.10	0.20	0.10	0.05	0.40	0.13	0.02	0.10	0.50	0.18	0.10	0.16	92
**PFBS**	0.10	0.20	0.06	0.05	0.20	0.11	0.05	0.10	0.50	0.15	0.10	0.14	94
**PFPeS**	0.10	0.20	NA	0.05	0.25	0.12	NA	0.10	0.50	0.19	0.12	0.15	81
**PFHpS**	0.10	0.20	0.50	0.05	0.30	0.12	0.05	0.10	0.50	0.21	0.12	0.18	84
**PFNS**	0.10	0.20	NA	0.05	0.75	0.04	NA	0.10	0.50	0.25	0.10	0.27	110
**PFDS**	0.10	0.20	NA	0.05	0.40	0.07	0.05	0.10	0.50	0.18	0.10	0.17	95
**PFBA**	0.50	10.00	0.12	0.50	0.51	2.00	0.20	0.10	0.50	1.60	0.50	3.20	200
**PFPeA**	0.50	0.20	0.10	0.50	0.82	0.31	0.20	0.10	0.50	0.36	0.31	0.24	67
**PFHxA**	0.10	0.20	0.10	0.10	2.00	0.25	0.05	0.10	0.50	0.38	0.10	0.62	165
**PFHpA**	0.10	0.20	0.10	0.05	0.40	0.10	0.05	0.10	0.50	0.18	0.10	0.16	91
**PFDA**	0.10	0.20	0.10	0.05	0.30	0.07	0.05	0.10	0.50	0.16	0.10	0.15	92
**PFUnDA**	0.10	0.20	0.08	0.05	0.35	0.04	0.05	0.10	0.50	0.16	0.10	0.16	98
**PFTrDA**	0.50	0.20	0.54	0.50	0.25	0.03	0.10	0.10	0.50	0.30	0.25	0.21	69
**PFTeDA**	0.50	0.20	0.48	0.50	1.80	0.01	0.10	NA	0.50	0.51	0.49	0.56	109
**PFDoDA**	0.10	0.20	0.64	0.10	0.70	0.02	0.10	0.10	0.50	0.27	0.10	0.26	97
**instrument**	LC-MS/MS	LC-MS/MS	LC-MS/MS	LC-MS/MS	LC-MS/MS	LC-MS/MS	LC-MS/MS	LC-MS/MS	HRMS				

Table S16. Calculated z-scores

	z-score								
	lab code								
**analyte**	**1**	**2**	**3**	**4**	**5**	**6**	**7**	**8**	**9**
**l-PFOS**	NA	0.19	-0.55	-0.15	0.18	0.28	-0.07	-0.71	1.07
**br-PFOS**	NA	2.86	-0.51	0.39	-0.56	1.48	-1.32	-0.51	-1.05
**t-PFOS**	-1.27	1.25	-0.51	-0.02	0.11	0.51	-0.23	-0.65	0.81
**PFOA**	-1.00	1.55	0.33	-0.08	0.13	0.04	0.25	-1.09	0.25
**PFNA**	-1.26	2.65	NA	-1.26	0.10	0.10	0.95	-0.92	NA
**PFHxS**	-0.65	2.99	-0.25	-0.65	0.58	-0.09	0.14	-0.65	0.54
**sum EFSA-4 (lb)**	-1.14	1.43	-0.13	-0.05	0.14	0.31	0.02	-0.5	0.21
**PFBS**	-0.3	0.07	-0.58	-0.58	0.16	0.16	0.62	-0.49	1.63
**PFHpS**	-1.32	2.17	NA	-2.24	0.15	0.7	-0.04	0.51	NA
**PFDS**	-0.11	1.97	NA	-1.69	0.75	-0.72	-1.99	-0.38	2.16
**PFBA**	-0.77	-1.17	-0.64	0.57	0.39	0.6	-0.04	-0.31	1.47
**PFPeA**	-0.82	1.61	-0.82	0.54	0.65	-0.06	-2.91	-0.59	1.52
**PFHxA**	-0.3	2.16	-0.86	0.05	0.59	-0.44	-0.1	-0.55	0.56
**PFHpA**	-0.84	1.6	0.2	0.02	0.41	-0.1	-0.67	-0.69	0.54
**PFDA**	-0.85	2.67	-0.75	-0.96	0.91	0.18	0.6	-0.75	NA
**PFUnDA**	-0.59	2.02	-0.59	-1.41	1.04	0.06	0.71	-1.08	NA
**PFDoDA**	-1.58	2.44	NA	-0.38	0.47	-0.55	-0.04	0.39	0.39
